# Nanoscaled Lithium Powders with Protection of Ionic Liquid for Highly Stable Rechargeable Lithium Metal Batteries

**DOI:** 10.1002/advs.201901776

**Published:** 2019-10-14

**Authors:** Kaichao Pu, Xiaolei Qu, Xin Zhang, Jianjiang Hu, Changdong Gu, Yongjun Wu, Mingxia Gao, Hongge Pan, Yongfeng Liu

**Affiliations:** ^1^ State Key Laboratory of Silicon Materials and School of Materials Science and Engineering Zhejiang University Hangzhou 310027 China; ^2^ Science and Technology on Aerospace Chemical Power Laboratory Hubei Institute of Aerospace Chemotechnology Xiangyang 441003 China

**Keywords:** anode materials, batteries, cryomilling, energy materials, lithium metal

## Abstract

To suppress the dendrite formation and alleviate volume expansion upon striping/platting is a key challenge for developing practical lithium metal anodes. Lithium metal in powder form possesses great potential to address this issue due to large specific surface area. However, the fabrication of powdery metallic lithium is largely restricted because of its unique softness, stickiness, and high reactivity. Here, a safe and readily accessible cryomilling process toward lithium powders is reported. Nanoscaled lithium powders (<500 nm) are successfully prepared from lithium foils with the assistance of a high‐melting‐point ionic liquid under cryogenic temperature. The prepared lithium powder anode exhibits superior electrochemical properties in symmetric cells, including extraordinarily low yet stable overpotential (≈50 mV), ultrahigh area capacity (30 mAh cm^−2^), and good long‐term cyclability (1200 h) even cycling at high current density (10 mA cm^−2^). The powdery form of lithium also functions as a favorable prelithiation reagent for lithium‐free anodes (e.g., Si, SiO, and SnO_2_). The findings open up a new avenue for the real‐world application of lithium metal anodes for next‐generation lithium batteries.

## Graphical abstract

Nanoscaled lithium powders are successfully prepared by cryomilling lithium foil with ionic liquid [P_4444_]TFSI at cryogenic temperature. The prepared lithium powder anode exhibits superior electrochemical properties, including extraordinarily low yet stable overpotential, ultrahigh area capacity, and good long‐term cyclability.

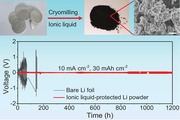

## 1 Introduction

Over the past few years, the rapidly growing demands for high energy density, high power density, and long cycle life Li‐ion batteries (LIBs) have stimulated tremendous efforts in exploring and developing novel electrode materials.^1^ As an ideal anode material, Li metal has an extremely high theoretical specific capacity of 3860 mAh g^−1^ and a low gravimetric density of 0.53 g cm^−2^ with the lowest negative electrochemical potential (−3.04 V vs standard hydrogen electrode).^2^ A theoretical energy density of 2600 Wh kg^−1^ for Li–S battery and 3500 Wh kg^−1^ for Li–air battery might be achieved with a Li metal anode.^3^ Practically, however, the use of Li metal anode in rechargeable batteries faces huge technical challenges in safety and short cycle life caused by the potential short circuit due to the Li‐dendrite growth and by the low coulombic efficiency (CE) due to the formation of unstable solid‐electrolyte interface (SEI) layer, respectively.^4^ Considerable effort has been devoted to the improvement of electrochemical performance of Li metal anode and development of rechargeable Li metal batteries.^5^ Attempts were made typically by the modification of electrolytes,^6^ or protection of Li metal anode,^7^ or use of advanced separators^8^ and solid‐state electrolytes.^9^ Nevertheless, most of these studies were based on thick dense Li foil, especially at laboratory level, which means economically not only the massive waste of Li source. The practical power operation of Li metal anode at high current density is also problematic due to the limited accessibility of the active surface of Li foil.^2^, ^3^, ^4^, ^5^, ^6^, ^7^, ^8^, ^9^ Although construction of 3D nanostructured Li hosts with graphene oxide,^10^ hollow carbon spheres,^11^ nanofiber matrix,^12^ and nanoporous scaffolds^13^ may be effective alternatives in homogenizing Li deposition and stabilizing SEI layer, the complicated manufacturing process of materials and electrodes, additional electrodeposition or thermal infusion process for prestoring Li into the hosts, in particular, is energy and time consuming, and consequently restricts their practical applications on the whole in battery manufacture industry.

Unlike Li foils, Li metal in powder form has advantages in the specific surface area and better compatibility with the existing battery manufacturing processes. In theory, the specific surface area of Li powder of ≈20 µm in diameter is 4.5 times higher than that of Li foil,^14^ which may substantially lower the local current density of the electrode, and hence decrease the polarization of Li anode. For the Li dendrite growth, multiple models have been developed, including Chazalviel model, Monroe–Newman model, Tikekar–Archer model, surface energy model, and phase field kinetics model.[qv: 5b,15–17] In Chazalviel model,^16^ it was demonstrated that the growth of Li dendrites depended strongly on the current densities. In particular, the initiation time of dendrite growth is inversely proportional to the square of the current density as described by the Sand's theory. We therefore believe that the Li dendrite growth may be suppressed while increasing the specific surface area. Moreover, the increased specific surface area is beneficial to reducing the volume expansion during the stripping and plating process of Li powder anode upon charging/discharging, thereby stabilizing the SEI layer.^16^ All these contribute significantly to the improved electrochemical performance and alleviated the safety risk.^14^, ^18^, ^19^, ^20^ In addition, the use of Li powders also allows a more controllable mass loading of Li, which is essential for a balanced mass of anode and cathode.

The current strategies for preparation of Li powders mainly focus on a melting dispersion and droplet emulsion technique.^14^, ^18^, ^19^, ^20^ The specific issue in question here is relying on high operating temperature, must be higher than the melting point of Li (>200 °C). In addition, the resulted Li powder particles are large in sizes, usually ranging from ≈20 to 50 µm.^21^ Therefore, facile and safe approaches to Li metal powders of smaller sizes are still highly desired. Unfortunately, no successful attempt has been reported so far, possibly due to the intrinsic soft, sticky, and chemically reactive characteristics of the metal, which prevent from making stable and readily handling Li particles in micrometer or smaller scale.

In this work, we demonstrate, for the first time, a safe and convenient approach to nanoscaled Li metal powders by means of cryomilling with a high‐melting‐point ionic liquid as a protection measure for Li during the milling process. Ball milling is a conventional technique for pulverization, in which the collisions between the grinding balls cause fracturing and cold welding of the materials particles thus reducing the particle/grain size, creating cracks, and fresh surfaces, and even producing some degree of alloying.^22^ However, traditional ball milling technique fails in preparing Li metal powder at ambient temperature because of the unique soft and sticky nature of the metal. Encouragingly, Li becomes brittle and hard, and processable at cryogenic temperature. Moreover, the high‐melting‐point ionic liquid also plays a critically important role by working as a milling assistor as well as a protective and dispersion agent due to its solid state feature at room temperature, which facilitates the formation of small‐scaled particles. The prepared Li powders measuring <500 nm in diameter not only exhibit superior electrochemical properties while assembling in symmetric cells, including remarkably low local current density and good long‐term cyclability, but also function as a favorable prelithiation reagent for Li‐free anodes (e.g., Si, SiO, and SnO_2_) to achieve high first CE. While cycling at a high current density of 10 mA cm^−2^, an overpotential as low as ≈50 mV with a capacity of 30 mAh cm^−2^ was observed after cycling 1200 h (200 cycles) without dendrite growth; this is largely superior to most of previous reports. Our findings in this work provide a new avenue for promoting the practical applications of Li metal in high‐energy density rechargeable batteries. It is also helpful to prepare small powders of other highly reactive soft metals, including Na, K, and so on.

## 2 Results and Discussion

### 2.1 Preparation and Characterization of Nanoscaled Li Powders

**Figure 1:**
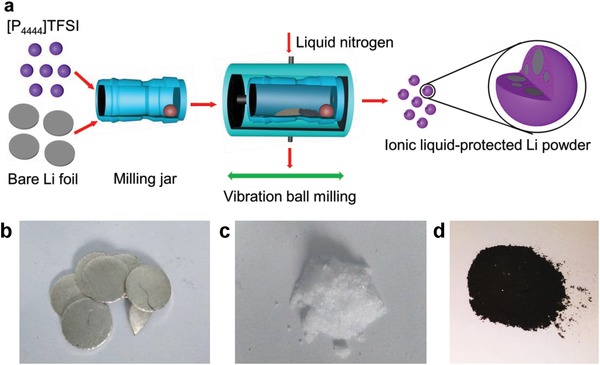
a) Schematic illustration of preparation process and optical images of b) bare Li foil, c) ionic liquid [P_4444_]TFSI, and d) cryomilled product.

**Figure** **1**a schematically illustrates the ionic liquid‐assisted cryomilling preparation process for nanoscaled Li powders. In order to prevent Li agglomeration and adhering to the surface of the milling vessel and balls and increase the milling efficiency, we applied an ionic liquid called tetrabutyl‐phosphonium bis(trifluoromethyl sulfonyl)imide, the melting point of which is 65 °C and chemical formula is C_18_H_36_F_6_NO_4_PS_2_ ([P_4444_]TFSI for short). Ionic liquids, a class of purely ionic, salt‐like materials, are liquid at low temperatures and have many unique properties such as ultralow volatility, good thermal stability, low flammability, tunable polarity and basicity/acidity, and ionic conductivity.^23^ In particular, there has been a significant amount of work recently demonstrating the benefit of using room‐temperature ionic liquids as electrolytes for Li metal batteries.^24^ Here, unlike room‐temperature ionic liquids, [P_4444_]TFSI ionic liquid is in solid state at room temperature, which functions as a dispersion agent, and at the same time as a protective agent for the fresh Li surface. The as‐received [P_4444_]TFSI ionic liquid was first heated and kept at 200 °C for 0.5 h in an Ar‐filled glove box to remove trace amount of H_2_O prior to use. Commercial Li foils were cut into small pieces of about 5 mm in size, and then loaded into a stainless steel grinding jar together with H_2_O‐free [P_4444_]TFSI at a mass ratio of 1:2 (molar ratio ≈ 1875:1). The ball milling was carried out on a Retsch CryoMill, with the grinding jar placed into an integrated autofill cooling system and continually cooled with liquid nitrogen to keep the temperature at −196 °C during the process. Unlike the bulk Li foils with metallic luster, dark powders were resulted after cryomilling at 20 Hz for 30 min (Figure 1b–d).

**Figure 2:**
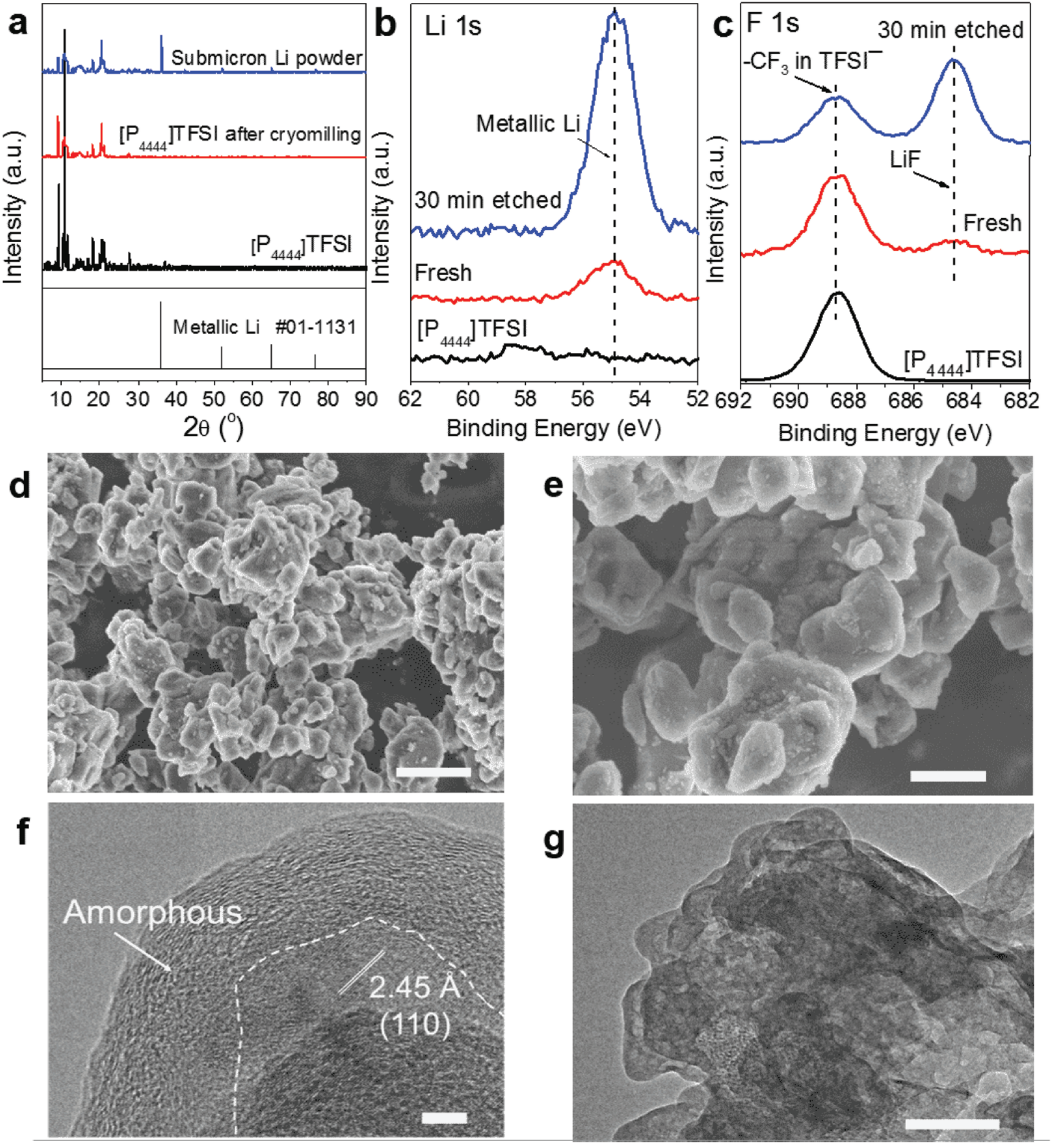
a) XRD patterns of cryomilled product, XPS spectra of b) Li 1s and c) F 1s of the prepared Li powders before and after 30 s sputtering and d,e) SEM and f,g) TEM images of ionic liquid‐protected nanoscale Li powders.

X‐ray diffraction (XRD) patterns of the dark powder from the cryomilling (**Figure** **2**a) displayed undoubtedly the characteristic reflections of metallic Li at 2θ = 36.2°, 52.2°, and 65.2° (JCPDS #01‐1131) along with weak reflections of [P_4444_]TFSI (2θ < 25°). The depth analyses of Li 1s X‐ray photoelectron spectroscopy (XPS) spectra revealed that much more metallic Li stayed in the depth than on the surface of resultant products (Figure 2b). Based on the XRD and XPS results, it seemed that the pulverized Li was covered by the [P_4444_]TFSI after cryomilling. Meanwhile, the Li—F bond of LiF species was identified from the F 1s XPS peak at 685.2 eV after 30 s Ar ion sputtering (Figure 2c).^25^ However, no appreciable change was observed in the O 1s XPS peak assignable to S=O groups of TFSI cations (Figure S1, Supporting Information).^26^ This indicates that most of [P_4444_]TFSI still persisted after cryomilling with metallic Li. To characterize the morphology of the Li metal particles, the cryomilled product was washed by cyclohexane to remove most of [P_4444_]TFSI. Well‐defined particle morphology was then observed with <500 nm in size (Figure 2d,e). The specific surface area of the Li particles was determined by Brunauer–Emmet–Teller (BET) experiment to be ≈ 5.257 m^2^ g^−1^ (Figure S2, Supporting Information), which is three orders of magnitude higher than that of Li foil (≈0.0023 m^2^ g^−1^). Transmission electron microscopy (TEM) observation further confirmed that metallic Li particles were covered by [P_4444_]TFSI (Figure 2f,g), which is in excellent agreement with the XRD and XPS results. Thus, cryomilling of Li foils with [P_4444_]TFSI ionic liquid yielded particle‐like Li powders sized <500 nm, in which Li metal was covered by [P_4444_]TFSI. Here, the high‐melting‐point ionic liquid [P_4444_]TFSI played critical roles as protection and dispersion agent, in favor of fine particle formation and final product collection and handling.

### 2.2 Electrochemical Performance of Nanoscaled Li Powder Electrodes

**Figure 3:**
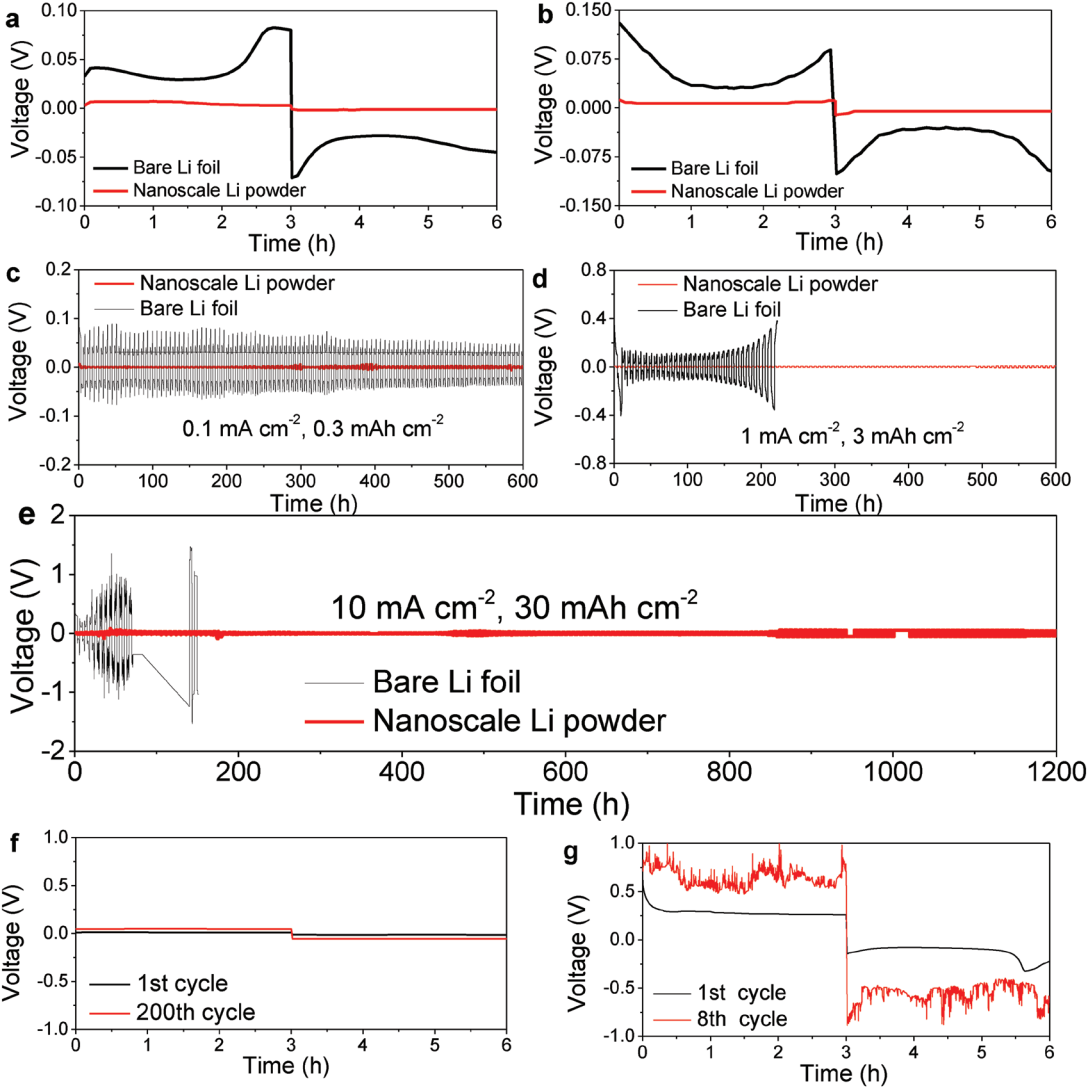
Voltage hysteresis for bare Li foil and nanoscale Li powder electrode at current densities of a) 0.1 mA cm^−2^ and b) 1 mA cm^−2^, cycling voltage profiles of bare Li foil and nanoscale Li powder electrode at current densities of c) 0.1 mA cm^−2^, d) 1 mA cm^−2^, and e) 10 mA cm^−2^, and voltage hysteresis of f) nanoscale Li powder electrode and g) bare Li foil electrode at selected cycles.

The ionic liquid‐protected Li powders in nanoscale were fabricated into wafer electrodes by a typical slurry method, and then assembled into 2025 coin‐type symmetric cells to evaluate the electrochemical plating/stripping performance at current densities of 0.1–10 mA cm^−2^. For reference, the bare Li foils were directly assembled into symmetric cells with the same separator and electrolyte. Prior to use, the surface oxide layer of Li foils was cleaned up (Figure S3, Supporting Information). It is clear from **Figure** **3**a,b that the nanoscaled Li powder electrode delivered very flat voltage plateaus with only 2.8 and 7.6 mV of voltage hysteresis in the initial cycle, while cycling at 0.1 and 1.0 mA cm^−2^, respectively, which were much lower than those of the bare Li foil electrode (≈34 mV at 0.1 mA cm^−2^ and 60 mV at 1.0 mA cm^−2^). As expected, much better cycling stabilities were observed for the nanoscaled Li powder electrode (Figure 3c,d), because the smaller the voltage hysteresis is, the less the electrode polarization will be. Even cycled at a very high rate of 10 mA cm^−2^ under 30 mAh cm^−2^ of area capacity density, the nanoscaled Li powder electrode still exhibited an excellent long‐term cyclability: up to 1200 h (200 cycles) (Figure 3e) with only a slight increase in voltage hysteresis (<50 mV) (Figure 3f). It is worth highlighting that the area capacity density of 30 mAh cm^−2^, corresponding to ≈8.3 mg cm^−2^ of Li loading density and the depth of discharge reached to nearly 85%, applied here is significantly higher than those of most of presently known Li metal electrodes, which surpasses the requirements in the real‐world where the electrode area capacity is normally in the range of 3–4 mAh cm^−2^.^27^ Moreover, the narrow and stable voltage hysteresis feature upon cycling is superior to all other Li metal electrodes reported so far while operating at identical current density (Figure S4 and Table S1, Supporting Information). Such favorable electrochemical performance is closely related to the significantly enlarged specific surface area of particle‐like Li powders because of the reduced area current density, which retards the nucleation of Li dendrites as indicated by the flat voltage hysteresis (Figure 3f). The specific capacity was determined to be 1250 mAh g^−1^ based on the total anode composite, which was further confirmed by pasting onto Cu foil (Figure S5a,b, Supporting Information). Also, the specific capacity can be increased to 3274 mAh g^−1^ after washing away the surface coated ionic liquid (Figure S5c, Supporting Information). While using the Cu foil as counter electrode, the CE was determined to be nearly 95% after 50 cycles, which was superior to Li foil (61%) (Figure S6, Supporting Information). By contrast, an obvious polarization was observed for the bare Li foil electrode even in the initial stage of cycling as there was a rapid increase in the voltage hysteresis from 258 to 790 mV (Figure 3g). After 20 cycles (120 h), an abrupt drop and severe fluctuation appeared in the voltage profile due to possible internal short circuit as reported previously.^28^

**Figure 4:**
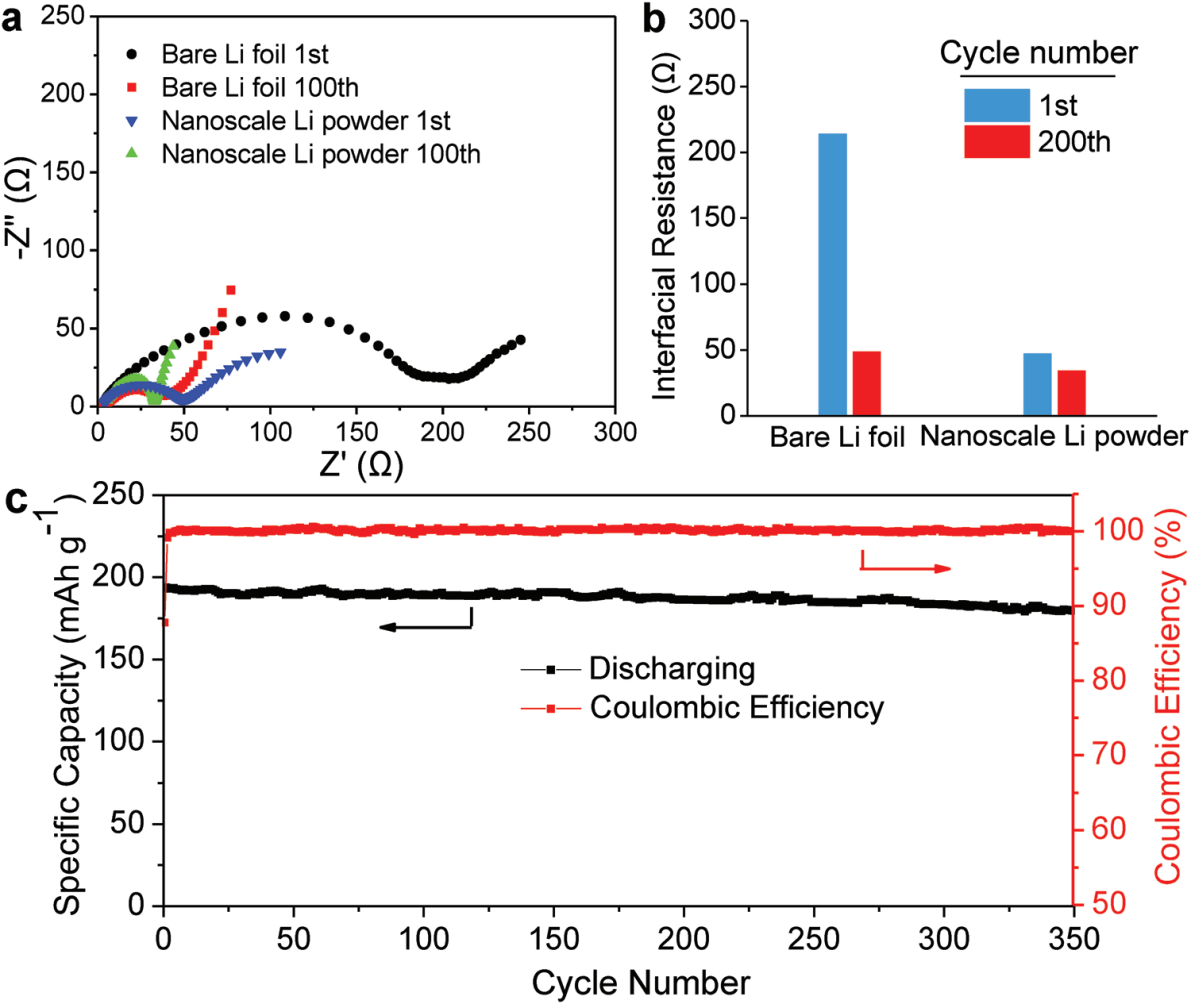
a) EIS spectra and b) the corresponding interfacial resistance values of bare Li foil electrode and nanoscale Li powder electrode at selected cycles, and c) cycling performance of nanosacle Li‐NMC 811 full cells.

Electrochemical impedance spectroscopy (EIS) examination further revealed a stable SEI film for the cycled Li powders since the interfacial resistance remained nearly constant after 100 cycles (**Figure** **4**a). Based on the equivalent circuit in Figure S7 (Supporting Information),^29^ the interfacial resistance was calculated to be 47.1 Ω after 1 cycle and 34.3 Ω after 100 cycles for the Li powder electrode (Figure 4b). The errors of data fitting are <5% as shown in Table S2 (Supporting Information). However, a dramatic reduction in the interfacial resistance was observed from 214 to 48.7 Ω with cycling for the bare Li foil electrode, which results in a very similar EIS spectrum with the Li powder electrode after 100 cycles. These can be attributed to two factors, including the exposure of fresh surface caused by the decomposition of SEI layer and the increased surface area caused by the Li dendrite growth during the Li stripping/plating processes.

A full cell was also assembled by using ionic liquid‐protected nanoscale Li powder as anode and LiNi_0.8_Mn_0.1_Co_0.1_O_2_ as cathode, denoted as Li‐NMC811, to further evaluate electrochemical properties. The initial charge and discharge capacities of the Li‐NMC811 full cell were determined to be 213 and 194 mAh g^−1^, respectively, while operating at charging and discharging rate of 0.2 C (41 mA g^−1^). After 350 cycles, the specific capacity still remained at 180 mAh g^−1^ with 92.7% of capacity retention and 99.9% of average CE, as shown in Figure 4c, indicating a reasonable long‐term cycling stability. Furthermore, variable rate cycling measurements delivered specific capacity of 180 mAh g^−1^ at 0.5 C, 161 mAh g^−1^ at 1 C, 143 mAh g^−1^ at 2 C, and 121 mAh g^−1^ at 5 C (Figure S8, Supporting Information), which are comparable with the Li foil‐NMC811 cells reported previously.^30^

### 2.3 Mechanism for Improved Electrochemical Properties

**Figure 5:**
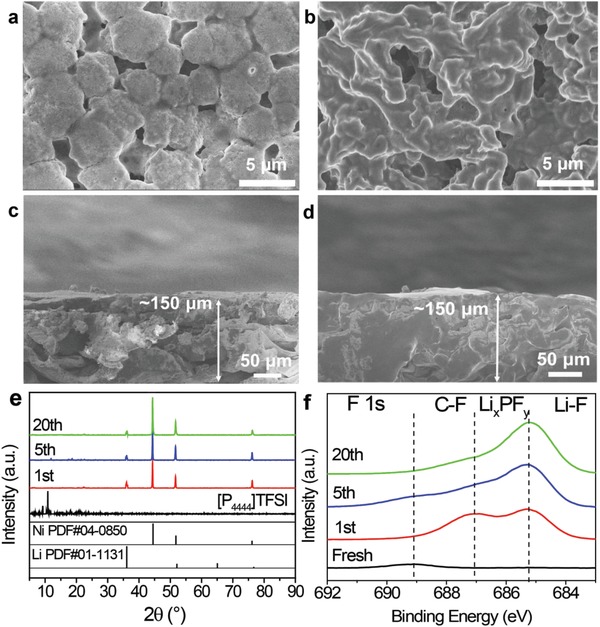
a,b) SEM images and c,d) cross‐sectional view images of electrodes fabricated from nanoscale Li powders (a,c) before and (b,d) after 20 cycles, and e) XRD patterns and f) XPS spectra of Li powder electrodes after various cycles.

**Figure 6:**
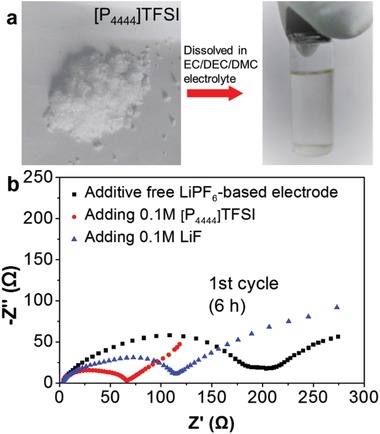
a) Optical images of [P_4444_]TFSI and the corresponding EC/DEC/DMC solution, b) EIS curves of symmetric cells with [P_4444_]TFSI and LiF as electrolyte additives.

To understand the good cycling stability of the nanoscaled Li powders, the cycled wafer electrodes were disassembled from the symmetric cells and washed by diethyl carbonate (DEC) for morphology observation and structural characterization. Unlike the Li foil electrode (Figure S9a,b, Supporting Information), the electrode surface of Li powder electrode looked still smooth and dense in morphology, and no Li dendrites were discernible after cycling, as shown in **Figure** **5**a,b, suggesting a uniform deposition of Li on the surface of the electrode and effective suppression of the Li dendrite growth. Moreover, the volume change was also effectively suppressed for the cycled Li powder electrode because it still remained relatively dense with the nearly unchanged thickness (Figure 5c,d). By contrast, the cycled Li foil electrode became quite loose and breakable (Figure S9c–f, Supporting Information), possibly due to the infinite volume change. These improvements are mainly ascribed to the larger surface area of Li powders, which largely lowered the local current density of the electrode surface and increase the utilization of active Li. XRD examination revealed that metallic Li still persisted after cycling for the Li powder electrode (Figure 5e). Meanwhile, the F 1s XPS peak of LiF maintained nearly constant after 5 cycles (Figure 5f), representing a stable SEI layer. Interestingly, the characteristic XRD reflections and XPS peaks assignable to [P_4444_]TFSI were invisible, due to the fact that it is soluble in the ethylene carbonate/diethyl carbonate/dimethyl carbonate (EC/DEC/DMC)‐based organic electrolyte (**Figure** **6**a). The dissolved [P_4444_]TFSI bearing TFSI^−^ anions remarkably facilitates the formation of a stable SEI layer on the surface of Li particles^31^ by working as an electrolyte additive together with the in situ formed LiF during cryomilling. Such conjecture was supported by directly adding [P_4444_]TFSI or LiF into the LiPF_6_‐based electrolyte, which largely reduced the interface resistance of Li metal anode, as shown in Figure 6b. Here, the high‐ and middle frequency regions corresponding to interface resistance seem to consist of two merged semicircles due to the joint contributions from SEI layer resistance as well as charge‐transfer resistance between electrode and electrolyte, as reported extensively.^29^ Instead, the cycled Li foil electrode exhibited a vast reduction in the peak intensities of metallic Li. The reflections of Li*_x_*PF*_y_* (2θ = 19.0°, 20.9°, 21.9°), Li_3_PO_4_ (2θ = 16.9°, 17.3°, 23.1°, 24.7°), and other organic species (2*θ =* 11°–15°), which are generally regarded as the major components of the typical SEI layer in the LiPF_6_‐based electrolyte, intensified with cycling (Figure S10, Supporting Information). Moreover, the peak intensity of F 1s XPS for LiF increased constantly upon cycling (Figure S11, Supporting Information), representing an unstable SEI layer due to the destroy of electrode surface (Figure S9, Supporting Information), although the fluoroethylene carbonate (FEC) was employed as the additive of electrolytes which facilitates the formation of SEI layer.^32^

### 2.4 Prelithiation Function of Nanoscaled Li Powders

**Figure 7:**
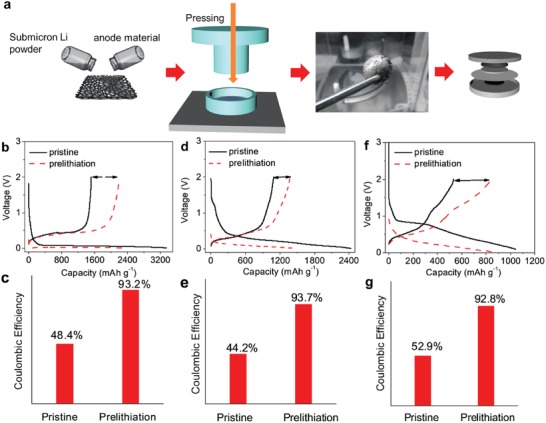
a) Schematic illustration of the utilization of nanoscale Li powder for prelithiating Li‐free anode materials, and b,d,f) the first discharge/charge voltage curves and c,e,g) first coulombic efficiency of (b,c) Si, (d,e) SiO, and (f,g) SnO_2_ before and after prelithiation.

Prelithiation is well known as a powerful approach to compensate for the first‐cycle irreversible capacity loss of anode materials caused by the formation of an SEI layer.^33^ However, the only prelithiation reagent in powder is stabilized Li metal powders with 20–50 µm in sizes, which are quite difficult to synthesize and handle, as pointed out by Cui and co‐workers.^34^ Here, we also examined the prelithiation capability of the sub‐micrometer Li powders by combining with Li‐free anode materials, including Si, SiO, and SnO_2_. The amount of the nanoscaled Li powders to be loaded was determined by matching the theoretical reversible capacity on the first cycle of the Li‐free anode materials, which was then cold‐pressed with the Li‐free anode materials onto a foam nickel current collector to fabricate the electrode (**Figure** **7**a). Figure 7b–g shows the first discharge/charge voltage profiles and the corresponding CE. As can be seen, a remarkable increase in the first charge capacity was observed for the prelithiation samples in comparison with the pristine samples. The first CE values calculated were increased from 48.4%, 44.2%, and 52.9% to 93.2%, 93.7%, and 92.8% for Si, SiO, and SnO_2_ anodes, respectively, comparable to that of commercial graphite anode.

## 3 Conclusion

In summary, we could fabricate 500 nm small Li metal particle composite by cryomilling Li foil with ionic liquid [P_4444_]TFSI, which was stable and easy handling. The Li powder anode delivered excellent electrochemical properties of very low overpotential and ultrahigh area capacity, and the Li‐dendrite growth could be effectively suppressed or prevented. A full cell constructed with the lithium powders as anode exhibited highly stable cyclability. Moreover, the ionic liquid protected‐nanoscale Li powders can also function as an effective prelithiation reagent for the compensation for the large irreversible capacity loss of Li‐free anode materials. Therefore, the realization in the preparation of stable Li powders in nanoscale opened up the new opportunity to use Li metal as anode material in the practice for the LIBs. Such top‐down strategy can be readily adopted for preparation of small‐sized particles from other highly reactive soft metals (e.g., Na and K) as well.

## 4 Experimental Section

*Preparation of Nanoscaled Li Powders*: The handling of all samples was conducted in a glove box (Etelux, China) filled with argon gas (H_2_O and O_2_: <0.1 ppm). The ionic liquid, tetrabutyl‐phosphonium bis(trifluoromethyl sulfonyl)imide, abbreviated as [P_4444_]TFSI, and Li foils with purity >99% were purchased from Lanzhou Greenchem ILs (China) and Tianchenghe Technology (Shenzhen, China), respectively. The cryomilling treatment was carried out on a vibrational cryomill system (Retsch Ltd., Germany) equipped with an integrated cooling system, in which the grinding jar was continually immersed in liquid nitrogen and the temperature was kept at −196 °C. A stainless steel ball with a diameter of 16 mm was used, and the ball‐to‐sample weight ratio was ≈30:1. The vibrational frequency was alternately set at 25 Hz for 5 min and 5 Hz for 1.5 min after prefreezing for 15 min.

*Characterization*: XRD analysis was performed on a MiniFlex 600 X‐ray diffractometer (Rigaku, Japan) with Cu Kα radiation at 40 kV and 40 mA. XRD data were collected over 2θ = 5°–90° in step increment of 0.02°. XPS analysis was conducted with an ESCALAB 250Xi system (Thermo Scientific) with Al Kα radiation. The XPS data were calibrated using the adventitious C 1s signal at 284.8 eV as a reference. The elemental distribution in depth was obtained by the Ar^+^ sputtering of a spot on the sample surface with a diameter of Φ2.5 mm at a voltage of 15 kV and current of 10 mA for 30 min. A Hitachi‐S4800 microscope (Japan) and an FEI TecnaiG2 F20 S‐TWIN system were employed for SEM and TEM observation, respectively. The N_2_ adsorption/desorption isotherms of BET measurement were collected at 77 K, with the relative pressure ranging from 0.0424 to 0.987 (P/P_0_) using a NOVA‐1000e automated surface area analyzer (Quantachrome, USA).

*Electrochemical Measurements*: The prepared Li powders were assembled into 2025 coin‐type symmetric cells as anode materials to evaluate the electrochemical properties by a galvanostatic charge–discharge technique. The wafer electrodes were fabricated by using a typical slurry method with 2 wt% poly(vinylidene fluoride (PVDF) solution as the binder. The slurry was pasted onto nickel foam and dried under Ar atmosphere in the glove box. For symmetric cells, ≈40 mg of Li powder composited was used, which contains ≈13 mg of active Li with loading density of 9.75 mg cm^−2^. A solution of 1 m LiPF_6_ with EC/DEC/DMC (1:1:1 by volume) and 1 vol% FEC was used as the electrolyte, and a Celgard 2400 membrane was used as the separator. The cells were galvanostatically cycled at given current densities (0.1, 1, and 10 mA cm^−2^) with a Neware battery testing system (Shenzhen, China) at constant temperature of 26 ± 1 °C. EIS results were recorded with an Ivium Vertex electrochemical workstation (The Netherlands) using an amplitude of 5 mV. In a full cell, the ionic liquid‐protected nanoscale Li powders were used as anode and commercial NMC as cathode. Approximately 180 mg of Li powder composite, which is composed of 60 mg of active Li (loading density: 45 mg cm^−2^) was weighed for anode and 28 mg of LiNi_0.8_Mn_0.1_Co_0.1_O_2_ for cathode (loading density: 20.5 mg cm^−2^). The full cell is cathode limited. For prelithiation process, the commercially purchased Si, SiO, and SnO_2_ powders were first milled with acetylene black in a weight ratio of 3:1 for 3 h, and then uniformly spread onto a nickel foam current collector with an area of 1.33 cm^2^. After that, the ionic liquid protected nanoscale Li powders were loaded on the Si, SiO, and SnO_2_ electrodes, which were cold‐pressed under a pressure of ≈10 MPa to fabricate the target electrodes.

## Conflict of Interest

The authors declare no conflict of interest.

## Supporting information

Supplementary
