# Supplementary material for: Nanoscaled Lithium Powders with Protection of Ionic Liquid for Highly Stable Rechargeable Lithium Metal Batteries
Source: Adv Sci (Weinh). 2019 Oct 14;6(24):1901776. doi: 10.1002/advs.201901776 (PMC6918098; doi:10.1002/advs.201901776)
Supplement: Supplementary file 1 — Supplementary [file ADVS-6-1901776-s001.pdf]

Copyright WILEY-VCH Verlag GmbH & Co. KGaA, 69469 Weinheim, Germany, 2019.

ADVANCED SCIENCE
Open Access

Supporting Information

for *Adv. Sci.,* DOI: 10.1002/advs.201901776

Nanoscaled Lithium Powders with Protection of Ionic Liquid for Highly Stable Rechargeable Lithium Metal Batteries

*Kaichao Pu, Xiaolei Qu, Xin Zhang, Jianjiang Hu, Changdong Gu, Yongjun Wu, Mingxia Gao, Hongge Pan, and Yongfeng Liu**

Copyright WILEY-VCH Verlag GmbH & Co. KGaA, 69469 Weinheim, Germany, 2019.

Supporting Information

Nano-scaled Lithium Powders with Protection of Ionic Liquid for Highly Stable Rechargeable Lithium Metal Batteries

Kaichao Pu, Xiaolei Qu, Xin Zhang, Jianjiang Hu, Changdong Gu, Yongjun Wu, *Mingxia Gao, Hongge Pan, and Yongfeng Liu**

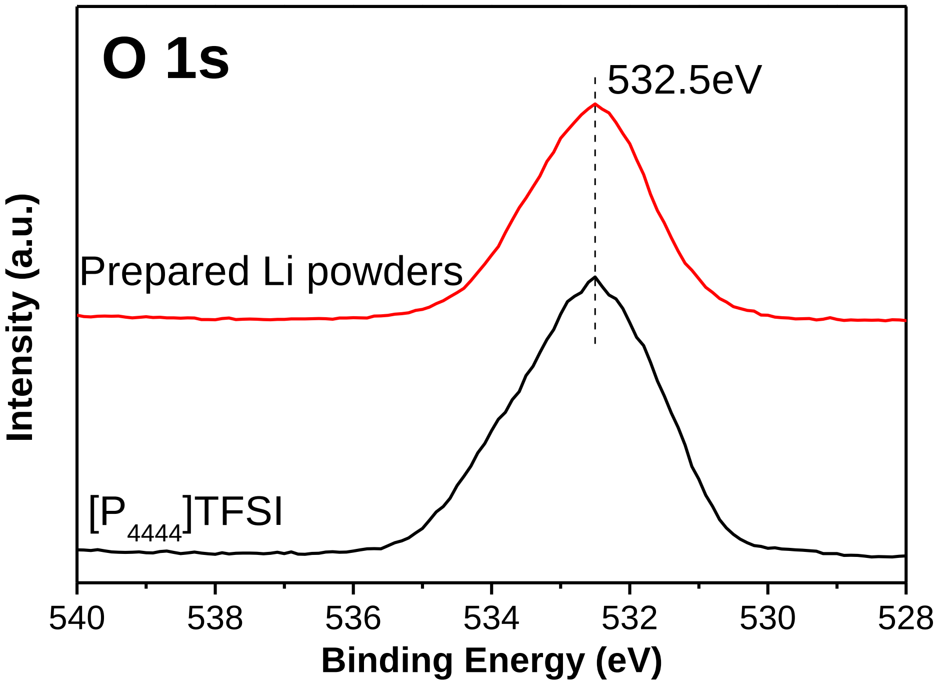

Figure S1 High-resolution O 1s XPS spectra of the ionic liquid $[P_{4444}]$TFSI and prepared Li powders.

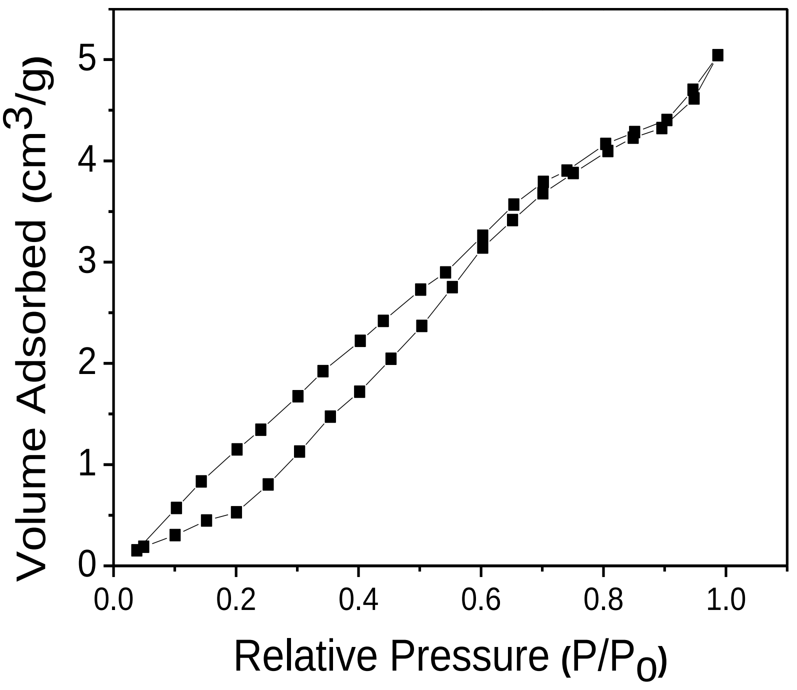

Figure S2 Nitrogen adsorption-desorption isotherms of ionic liquid protected Li powder sample (BET measurement).

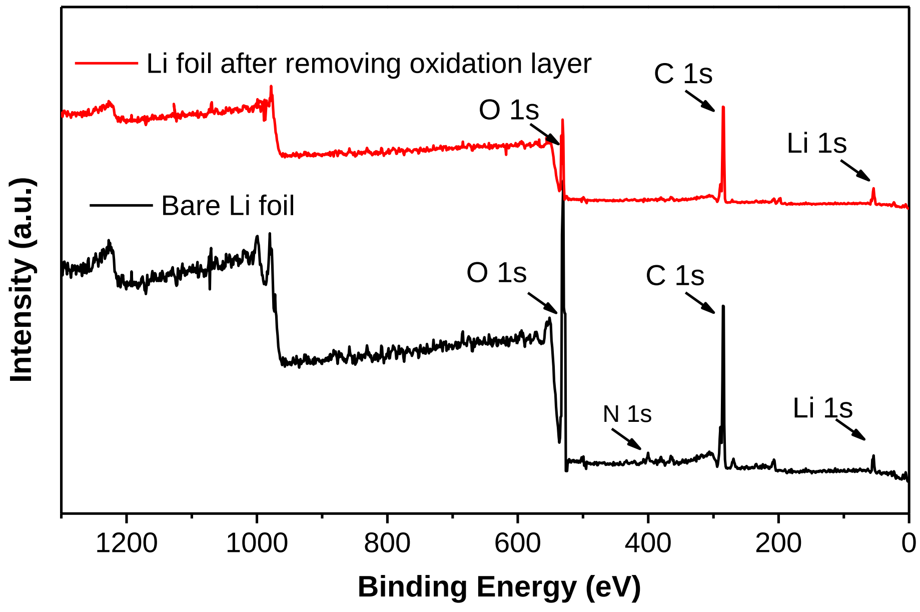

Figure S3 Survey XPS spectra of bare Li foil before and after removing oxidation layer

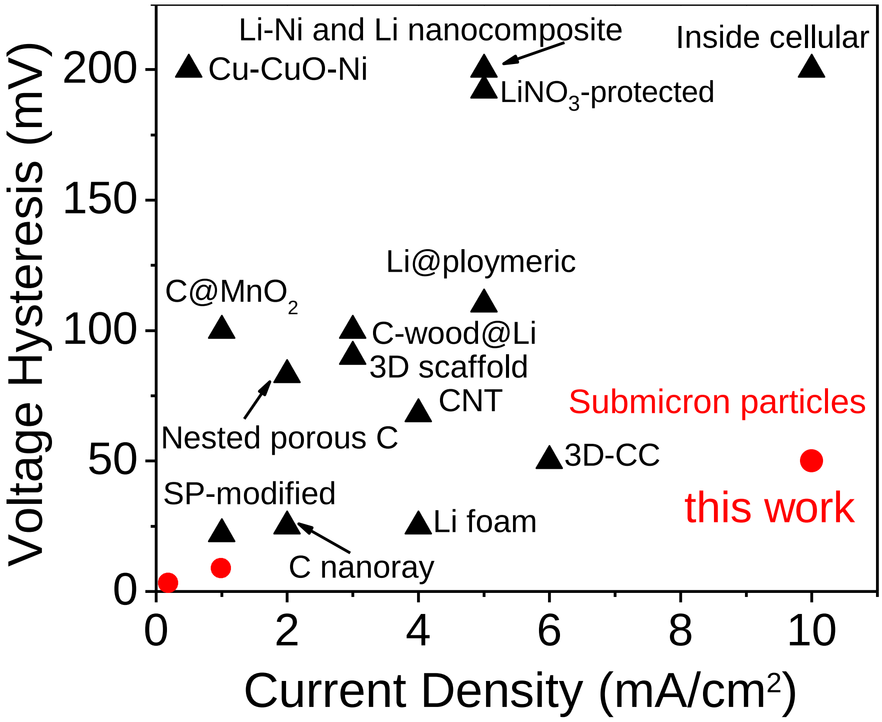

Figure S4 Voltage hysteresis and current density of Li metal anodes in this work and previous publications from other groups in recent years

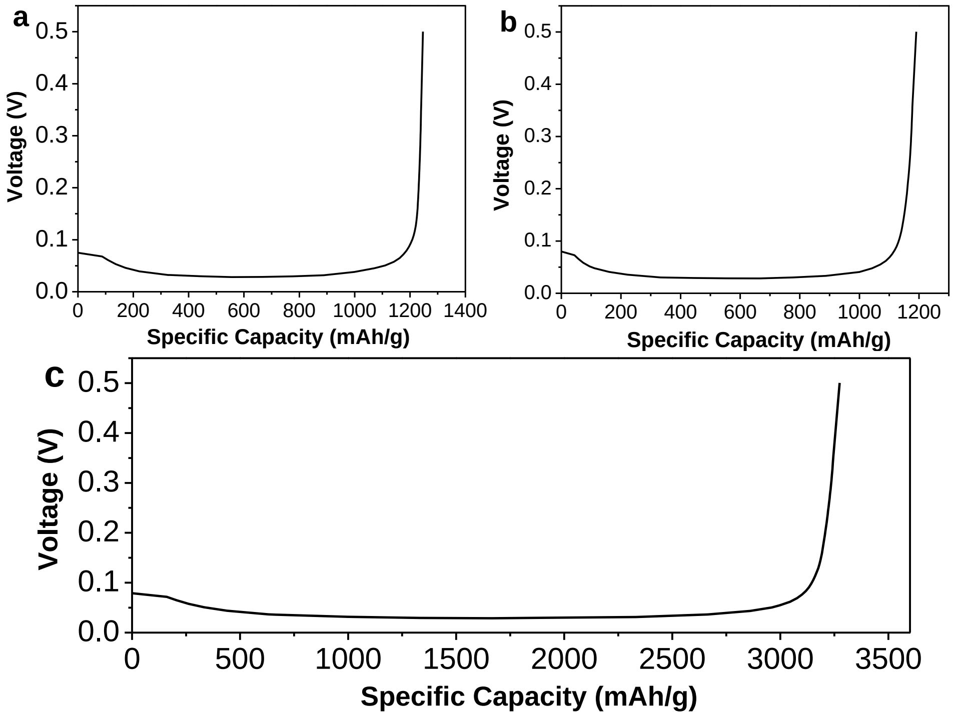

Figure S5 Specific capacity of nanoscale Li powder electrodes fabricated under different conditions. a) Pasted on Ni foam, b) pasted on Cu foil and c) after washing away the surface coated ionic liquid.

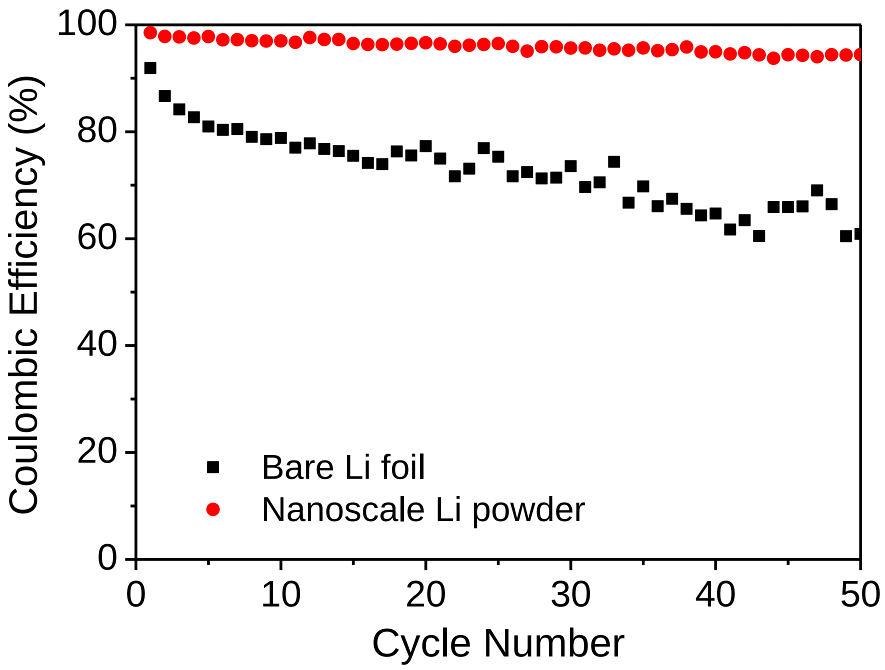

Figure S6 The Coulombic efficiency of bare Li foil and nanoscale Li powder electrode at 1 mA cm^{-2} for 1 mAh cm^{-2} as a function of cycles.

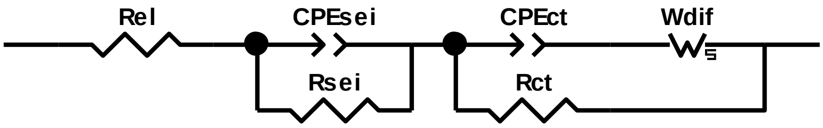

Figure S7 Equivalent circuit model used for fitting the impedance spectra.

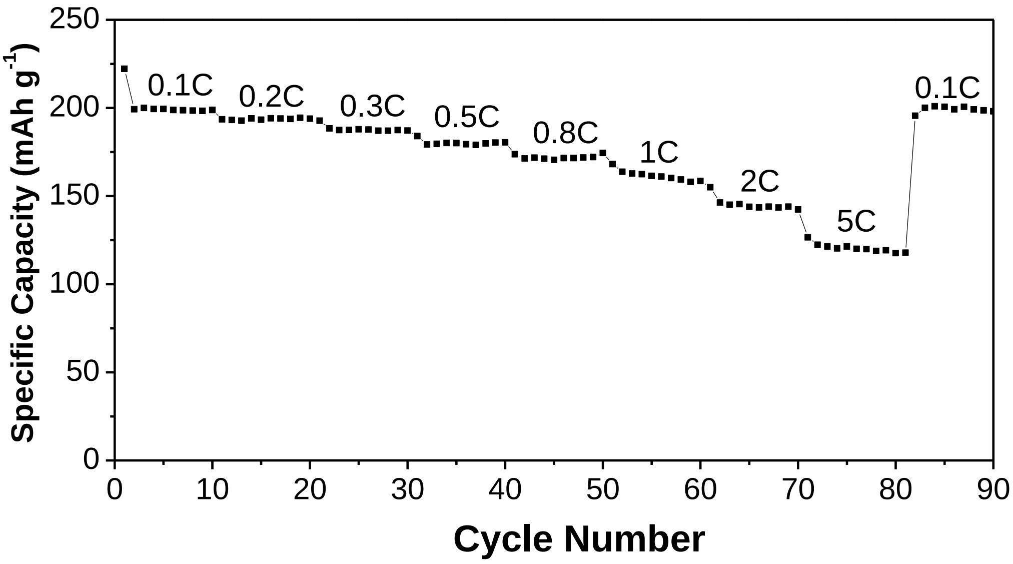

Figure S8 Rate performance of the nanoscale Li-NMC 811 full cell.

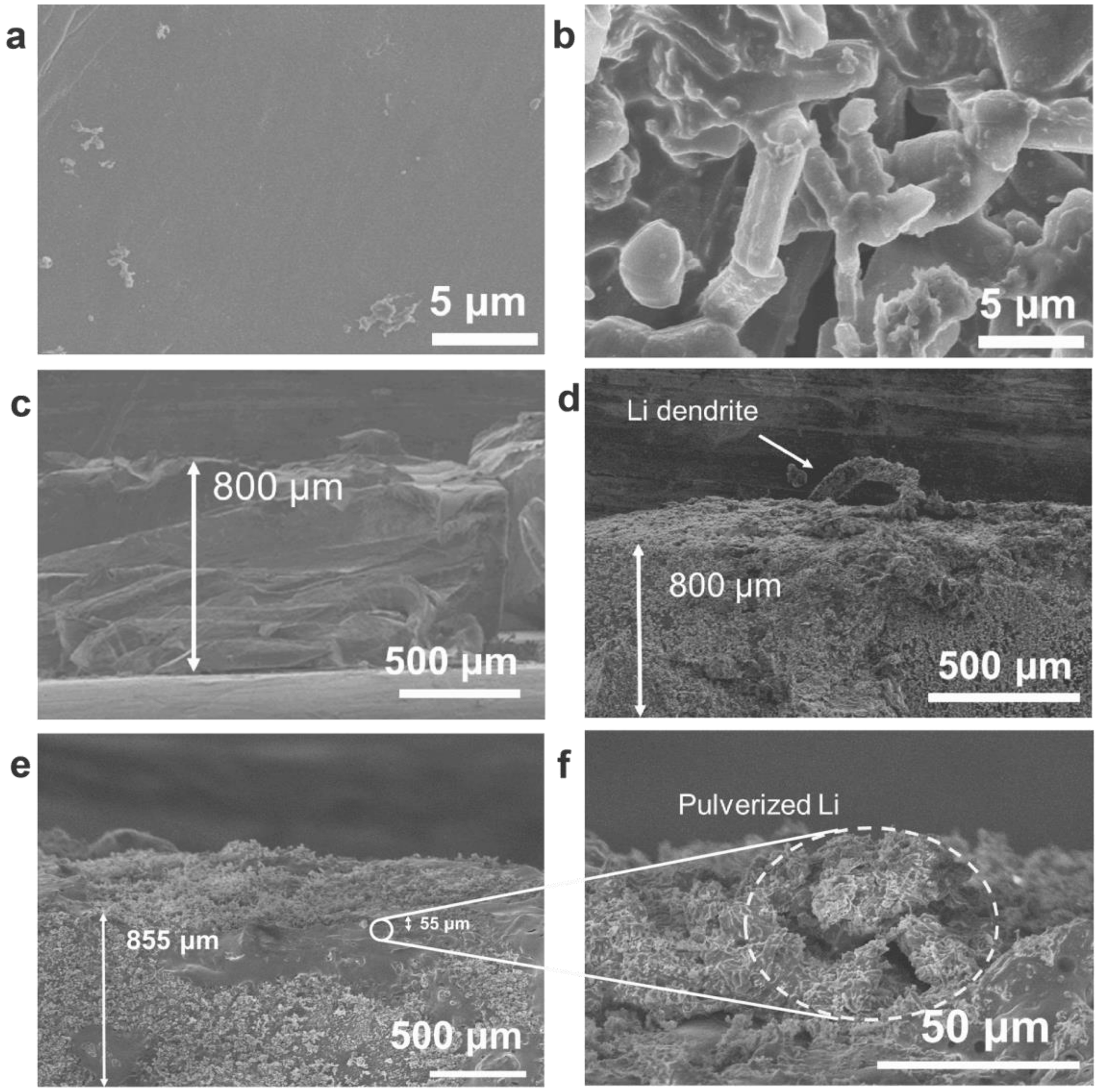

Figure S9 Top view of SEM images of bare Li foil (a) before cycling, (b) after 20 cycles, and cross-sectional view images of electrodes fabricated from bare Li foil (c) before cycling, (d) after 1 cycle, and (e, f) after 20 cycles.

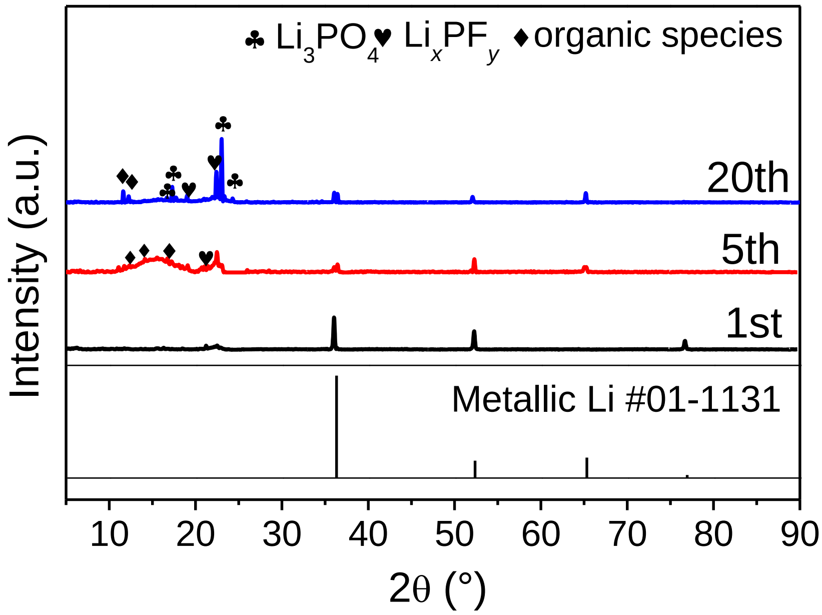

Figure S10 XRD patterns for bare Li foil electrodes after various charge/discharge cycles.

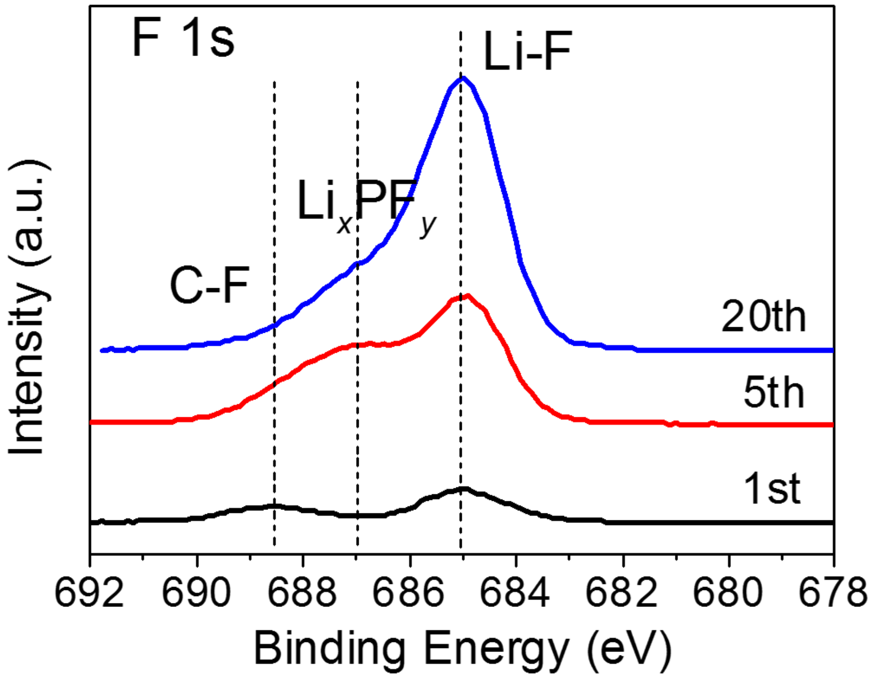

Figure S11 XPS spectra of F 1s of bare Li foil electrodes after various charge/discharge cycles.

Table S1 Voltage hysteresis comparison between this work and other recent publications.

Samples in **Figure S4**	Detailed description of samples	Current density (mA cm^{-2})	Overpotential (mV)	Cycle number (time)	Ref.
Nano-scaled Li particles	Prepared by cryo-milling	10	50	200 cycles (1200 h)	This work
3D scaffold	Infusion of Li into 3D conducting scaffold with lithiophilic	3	<90	80 cycles,	S1
Li@ploymeric	Lithium-coated polymeric matrix	5	~110	100 cycles	S2
C@MnO_2	CNT/Activated carbon nanofibers@MnO_2 electrode	1	~100	(450 h)	S3
Nested porous C	Graphene nested porous carbon current collector for LMBs	2	83	(500 h)	S4
CNT	Oxygen-rich CNT electrode	4	68	200 cycles	S5
$LiNO_3$-protected	$LiNO_3$-protected Li metal anode	5	192	(420 h)	S6
Cu-CuO-Ni	Lithiophilic Cu-CuO-Ni	0.5	~200	(580 h)	S7
C nanoray	Labber like carbon nanorays on 3D conducting skeletons	2	25	(1000 h)	S8
3D-CC	3D current collector with lithiophilic property	6	50	250 h	S9
Inside cellular	Li metal inside Cellular graphene scaffold	10	~200	(70 h)	S10
Li foam	porous Li foam	4	25	(80 h)	S11
N-C	Nitrogen-doped graphitic carbon foams	3	25	(1200 h)	S12
Li-Ni	Li-Ni composite electrode	5	200	(40 h)	S13
SP-modified	SP-modified electrode	1	22	120 cycle	S14
Li nanocomposite	Li-ion conductive nanoconposite electrode	5	~200	100 cycles	S15
C-wood@Li	Channel structure of C-wood infused by metallic Li	3	100	101 cycles	S16

Table S2` The fitted data of interfacial resistance and their error margins.

Samples	Fitted values of interfacial resistance (Ω)	Errors of fitting (%)
Nanoscale Li powder after 1 cycle	48.7	3.5
Nanoscale Li powder after 100 cycles	34.3	4.2
Bare Li foil after 1 cycle	214	2.8
Bare Li foil after 100 cycles	48.7	3.0

References

[S1] Z. Liang, D. Lin, J. Zhao, Z. Lu, Y. Liu, C. Liu, Y. Lu, H. Wang, K. Yan, X. Tao, Y. Cui, *Proc. Natl. Acad. Sci. USA* **2016**, *113*, 2862.

[S2] Y. Liu, D. Lin, Z. Liang, J. Zhao, K. Yan, Y. Cui, *Nat. Commun.* **2016**, *7*, 10992.

[S3] H. Xu, L. Xie, A. Manthiram, *Nano Energy* **2016**, *26*, 224.

[S4] W. Deng, W. Zhu, X. Zhou, Z. Liu, *Energy Storage Mater.* **2018**, *15*, 266.

[S5] K. Liu, Z. Li, W. Xie, J. Li, D. Rao, M. Shao, B. Zhang, M. Wei, *Energy Storage Mater.* **2018**, *15*, 308.

[S6] Q. Shi, Y. Zhong, M. Wu, H. Wang, *Proc. Natl. Acad. Sci. USA* **2018**, *22*, 5676.

[S7] S. Wu, Z. Zhang, M. Lan, S. Yang, J. Cheng, J. Cai, J. Shen, Y. Zhu, K. Zhang, W. Zhang, *Adv. Mater.* **2018**, *30*, 1705830.

[S8] L. Liu, Y. Yin, J. Li, Y. Guo, L. Wan, *Chem. Commun.* **2018**, *54*, 5330.

[S9] G. Yang, J. Chen, P. Xiao, P. O. Agbola, I. Shakir, Y. Xu, *J. Mater. Chem. A* **2018**, *6*, 9899.

[S10] W. Deng, X. Zhou, Q. Fang, Z. Liu, *Adv. Energy Mater*. **2018**, *8*, 1703152.

[S11] M. Hafez, Y. Jiao, J. Shi, Y. Ma, D. Cao, Y. Liu, H. Zhu, *Adv. Mater.* **2018**, *30*, 1802156.

[S12] L. Liu, Y. Yin, J. Li, S. Wang, Y. Guo, L. Wan, *Adv. Mater.* **2018**, *30*, 1706216.

[S13] S. Chi, Y. Liu, W. Song, L. Fan, Q. Zhang, *Adv. Funct. Mater.* **2017**, *27*, 1700348.

[S14] K. Liu, A. Pei, H. R. Lee, B. Kong, N. Liu, D. Lin, C. Liu, P. C. Hsu, Z. Bao, Y. Cui, *J.*

Am. Chem. Soc. **2017**, *139*, 4815.

[S15] D. Lin, J. Zhao, J. Sun, H. Yao, Y. Liu, K. Yan, Y. Cui, *Proc. Natl. Acad. Sci. USA* **2017**, *18*, 4613.

[S16] Y. Zhang, W. Luo, C. Wang, Y. Li, C. Chen, J. Song, J. Dai, E. M. Hilz, S. Xu, C. Yang, Y. Wang, L. Hu, *Proc. Natl. Acad. Sci. USA* **2017**, *14*, 3584.
